# 1028. Case Study. Case Review of Rehabilitation for Two High Voltage Electrical Injuries with Spinal Cord Injury Symptoms

**DOI:** 10.1093/jbcr/irag033.544

**Published:** 2026-04-06

**Authors:** Lily DuRose, Sarah Barth, Callie M Thompson

**Affiliations:** University of Utah Health, Salt Lake City, UT; University of Utah Health, Salt Lake City, UT; University of Utah Health, Salt Lake City, UT

## Abstract

**Patient Presentation (age range, injury details, relevant history):**

This paper reviews two cases with high voltage electrical injury. Case One: 73-year-old male with 14.5% TBSA full-thickness burns to head, neck, torso, BUE. Case Two: 24-year-old male with 69.5% TBSA partial & full-thickness burns to torso, L UE, B LEs, v-fib requiring shock & intubation.

**Clinical Challenges:**

Both patients exhibited early neurological deficits without radiologic confirmation. Case One: Initial deficits included L transradial amputation, posterior lean, poor proprioception, sensory loss, & inability to maintain midline. Case Two: L shoulder disarticulation, R AKA, sensory & motor deficits to R UE & L LE, required cultured epidermal autografting for definitive wound coverage.

**Management Approach:**

Intensive, high-frequency therapy was critical to both burn-specific & neurorehabilitation limitations. ICU-related weakness & endurance deficits were also incorporated.

**Outcomes:**

Case One: Required 62d in the Burn ICU & 3wks of inpatient rehabilitation. At discharge (PBD 83), he ambulated with assistive devices & continued therapy. At 3y post-injury he has full ROM & pliable scars & ambulates with forearm crutches on uneven terrain with mild ataxia. Case Two: Required 91d in Burn ICU & 61d in inpatient rehab. Pt regained some strength and was discharged home (PBD 145) with a power wheelchair, adaptive equipment, & ongoing outpatient neuro & burn rehab. Neither pt achieved full recovery, but both discharged home with significant functional gains.

**Lessons Learned:**

High voltage electrical injuries impact multiple systems & often present with unpredictable deficits. Neurologic symptoms with immediate or delayed onset consistent with SCI have been reported despite unremarkable imaging. This poses challenges in prognosis & goal setting. Burn scar maturation typically spans 12–18 m, aligning with optimal neuroplasticity timelines for neurologic recovery. Intensive rehabilitation during this window is crucial to maximize outcomes for burn & neurologic sequelae. Rehabilitation is critical to recovery, but limited literature exists outlining interventions or outcomes.

**Applicability to Practice:**

High voltage electrical injuries with neurological symptoms require early & aggressive rehabilitation. Absence of imaging findings does not exclude functional deficits; treatment should be symptom driven. Recovery potential mirrors burn scar & neuroplasticity timelines, with most progress in the first 12–18 m. Combined burn & neurorehabilitation can lead to meaningful improvement, though full resolution of neurological symptoms remains rare.

